# ABA mediates development-dependent anthocyanin biosynthesis and fruit coloration in *Lycium* plants

**DOI:** 10.1186/s12870-019-1931-7

**Published:** 2019-07-15

**Authors:** Gen Li, Jianhua Zhao, Beibei Qin, Yue Yin, Wei An, Zixin Mu, Youlong Cao

**Affiliations:** 1 National Wolfberry Engineering Research Center, Ningxia Academy of Agriculture and Forestry Sciences, Yinchuan, 750002 China; 2 College of Life Sciences, Northwest A&F University, Yangling, 712100 Shaanxi China

**Keywords:** *Lycium*, Fruit ripening, Anthocyanins, ABA, VIGS

## Abstract

**Background:**

Anthocyanins, which are colored pigments, have long been used as food and pharmaceutical ingredients due to their potential health benefits, but the intermediate signals through which environmental or developmental cues regulate anthocyanin biosynthesis remains poorly understood. Fleshy fruits have become a good system for studying the regulation of anthocyanin biosynthesis, and exploring the mechanism underlying pigment metabolism is valuable for controlling fruit ripening.

**Results:**

The present study revealed that ABA accumulated during *Lycium* fruit ripening, and this accumulation was positively correlated with the anthocyanin contents and the *LbNCED1* transcript levels. The application of exogenous ABA and of the ABA biosynthesis inhibitor fluridon increased and decreased the content of anthocyanins in *Lycium* fruit, respectively. This is the first report to show that ABA promotes the accumulation of anthocyanins in *Lycium* fruits. The variations in the anthocyanin content were consistent with the variations in the expression of the genes encoding the MYB-bHLH-WD40 transcription factor complex or anthocyanin biosynthesis-related enzymes. Virus-induced *LbNCED1* gene silencing significantly slowed fruit coloration and decreased both anthocyanin and ABA accumulation during *Lycium* fruit ripening. An qRT-PCR analysis showed that *LbNCED1* gene silencing clearly reduced the transcript levels of both structural and regulatory genes in the flavonoid biosynthetic pathway.

**Conclusions:**

Based on the results, a model of ABA-mediated development-dependent anthocyanin biosynthesis and fruit coloration during *Lycium* fruit maturation was proposed. In this model, the developmental cues transcriptionally activates *LbNCED1* and thus enhances accumulation of the phytohormone ABA, and the accumulated ABA stimulates transcription of the MYB-bHLH-WD40 transcription factor complex to upregulate the expression of structural genes in the flavonoid biosynthetic pathway and thereby promoting anthocyanin production and fruit coloration. Our results provide a valuable strategy that could be used in practice to regulate the ripening and quality of fresh fruit in medicinal and edible plants by modifying the phytohormone ABA.

## Background

The mechanisms underlying fruit development and ripening are important research hotspots due to their intimate relationship with fruit quality. Fruit maturation, which is a natural physiological process in the plant lifecycle, involves a series of physical, physiological and biochemical reactions [1]. Among these reactions, pigmentation resulting in coloration is a natural and excellent phenotype marker for the apparent degree of fruit maturity and can also act as a model system for studying the mechanism underlying the regulation of fruit ripening and coloration [2]. Anthocyanidins, which are considered flavonoid compounds, are water-soluble natural pigments that are widely found in plant petals and fruits. Anthocyanins produce red, blue and purple hues according to the pH value of the vacuole [3, 4]. It is generally recognized that anthocyanin accumulation in fruits is accompanied by fruit maturation and is regulated by both developmental and environmental cues. The structural genes involved in anthocyanin biosynthesis, which belongs to the classical flavonoid synthesis pathway [5], in the fruits of many plant species, such as tomatoes, cherries, strawberries and apples, have been identified and cloned [6–8]. In addition, previous studies have shown that anthocyanin biosynthesis is regulated mainly by the transcription factor MYB-bHLH-WD40 complex at both the transcriptional and posttranslational levels [9–11]. However, the link between developmental and environmental cues and these transcription factors remains unknown.

It is well known that phytohormones can also mediate anthocyanin biosynthesis by regulating the expression of genes involved in the flavonoid biosynthetic pathway. Abscisic acid (ABA), ethylene (Eth) and jasmonic acid (JA) are known to promote anthocyanin biosynthesis and enhance fruit ripening, whereas auxin and gibberellin (GA) inhibit anthocyanin production and thus delay fruit maturation [5, 12, 13]. ABA plays an important role in plant growth, stomatal movement, seed dormancy and germination, and the plant response to biotic and abiotic stress [14]. These ABA-mediated physiological processes are mainly affected by the regulation of the size of the bioactive ABA pool [15, 16]. The ABA biosynthesis pathway has been extensively studied and validated through molecular genetic approaches, and its key reaction is the oxidative cleavage of 9-cis-epoxyxanthophylls by 9-cis-epoxycarotenoid dioxygenase (NCED) to produce xanthoxin [14], which is an irreversible reaction and a rate-limiting step in ABA biosynthesis. Numerous studies have shown that ABA is generally involved in the regulation of fruit ripening in both climacteric and nonclimacteric fruits [2, 6, 16], in which the *NCED* gene plays an important role [8, 15, 17–23]. However, whether NCED-derived ABA is involved in anthocyanin-mediated fruit ripening in medicinal and edible plants remains poorly understood [24].

Wolfberry (*Lycium*, of the family Solanaceae) is a perennial, deciduous shrub growing in Northwest China and the Mediterranean region [25]. Its fruit, goji, has been used for centuries in China as a traditional herbal medicine and as a valuable nourishing tonic [26]. Recently, medical research has indicated that these fruits have many pharmacological functions, such as improving visual acuity, nourishing the liver and kidneys, reducing the blood sugar levels, reducing the risks of cancer and cell senescence and improving immunity [4]. Improvements in people’s living standards and their enhanced awareness of health-related issues are increasing the market demand for goji. In addition, people’s consumption patterns will gradually change from dried fruits to fresh fruits, as has been observed for the fruits of other fruit trees. In *Lycium* plants, both the fruit ripening time and the fruit quality regulation are not only a topic of basic research but also a key agronomic trait. However, compared with the study of fruits from other fruit trees, the study of ripening and coloration regulation in *Lycium* fruits remains scarce [27].

There exists a natural variation in the *Lycium* fruit color, and these plants are thus a good material for studying pigment metabolism [28]. It has been shown that anthocyanins are the dominant pigment in *Lycium* fruit with a mature black color (BF), whereas carotenoids are responsible for the coloration of *Lycium* fruit with a ripe red color (RF) [27, 29]. *Lycium* fruit with a yellow color (YF), which is another naturally occurring representative variety of *Lycium*, reportedly contains approximately one-tenth of the carotenoid level found in RF [30], which indicates that both anthocyanins and carotenoids are responsible for the coloration in ripe YF. For the sake of universality, *Lycium* fruits of two different colors, BF and YF, were used in this study to assess the relationship between anthocyanin biosynthesis and the ABA level during *Lycium* fruit ripening. Our aim was to link developmental cues with ABA signaling in anthocyanin biosynthesis and to provide a feasible strategy for the regulation of *Lycium* fruit ripening and quality that could be used in practice.

## Results

### Relationship between ABA and anthocyanins during *Lycium* fruit ripening

**Fig. 1:**
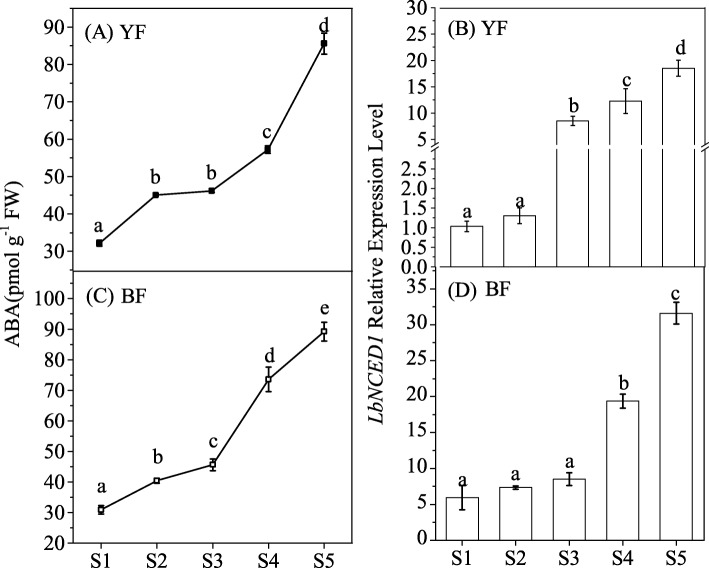
Time-course of ABA concentration (**a**, **c**) and *LbNCED1* transcript level (**b**, **d**) during *Lycium* fruit ripening under natural conditions. (**a**, **b**) Yellow-colored fruits (YF) and (**c**, **d**) blank-colored fruits (BF). The gene expression differences among the varieties were compared using the S1 stage of YF as the control for calculating the relative expression levels of the *LbNCED1* gene. The error bars represent the SDs of three independent replicates. Different letters on the bars for the same variety indicate significant differences between the treatments (*P* < 0.05)

*LbNCED1,* as the homologous sequence of *SlNCED1* in tomato and *StNCED1* in potato, was previously isolated from *Lycium chinense*, *Lycium barbarum* and *Lycium ruthenicum* [29, 31]. Because the relationship between the *LbNCED1* transcript amount and the ABA level has been determined in *Lycium chinense*, one of the red-colored *Lycium* fruits [31], the present study further detected this relationship in *Lycium* fruits with two other important colors, YF and BF. The results showed that the content of the endogenous hormone ABA and the *LbNCED1* gene expression level increased with fruit ripening in both colors of the fruits. Specifically, the content of ABA in YF increased from 32.17 ± 0.76 pmol·g^− 1^ FW during the S1 period to 85.57 ± 2.66 pmol·g^− 1^ FW during the S5 period, and the content of ABA in BF increased from 30.94 ± 1.36 pmol·g^− 1^ FW during the S1 period to 89.23 ± 2.88 pmol·g^− 1^ FW during the S5 period (Fig. 1a,c). The expression of the *LbNCED1* gene in YF increased 15.87-fold from the S1 period to the S5 period, whereas that in BF increased 4.88-fold from the S1 period to the S5 period (Fig. 1b,d). These results indicated that the expression of *LbNCED1* during fruit development and ripening might contribute universally to the accumulation of ABA in *Lycium* fruits.

**Fig. 2:**
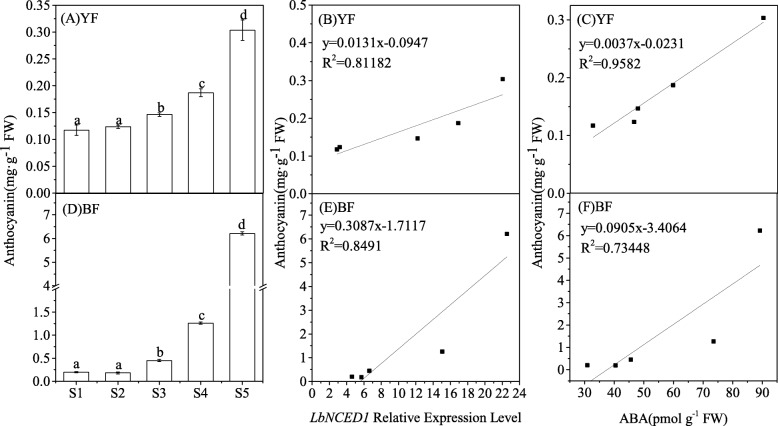
Time-course of the anthocyanin concentration (A, D) and the correlation between the anthocyanin content and *LbNCED1* transcripts (B, E) or ABA content (C, F) during *Lycium* fruit ripening under natural conditions. The error bars represent the SDs of three independent replicates. Different letters on the bars for the same variety indicate significant differences between the treatments (P < 0.05). “*” indicates P < 0.05, and “**” indicates *P* < 0.01

The content of anthocyanins in these two *Lycium* fruits was determined at the five stages (S1-S5) of the ripening process, and the content of this pigment significantly accumulated with increased fruit maturity (Fig. 2a, d). A detailed analysis of the dynamics in both fruits revealed that anthocyanins started to increase at stage S3 and peaked at stage S5. In addition, an approximately 20-fold difference was found between the maximal anthocyanin content of BF and that of YF. It is indicated that anthocyanin levels were affected both by developmental stage and genotype in *Lycium* fruits. In this study, a linear correlation analysis was performed between the anthocyanin content, and the *LbNCED1* gene transcript (Fig. 2b, e) or the ABA level (Fig. 2c, f) in these two different fruits, and the results revealed the existence of a significantly positive linear relationship between anthocyanin and ABA in *Lycium* fruits, irrespective of their color.

### Effects of exogenous ABA treatment and inhibition of endogenous ABA synthesis on anthocyanin biosynthesis in *Lycium* fruit

**Fig. 3:**
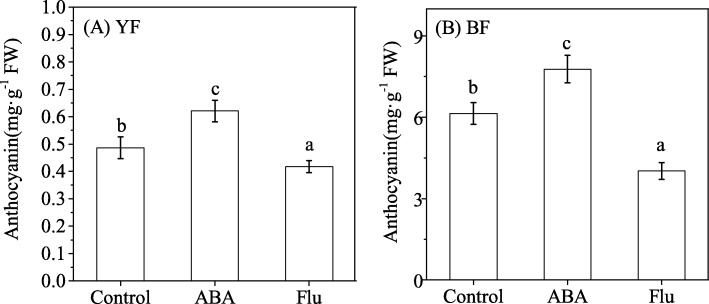
Effect of exogenous ABA or ABA biosynthesis inhibitor fluridon (Flu) on anthocyanin content. The error bars represent the SDs of three independent replicates. Different letters on the bars for the same variety indicate significant differences between the treatments (P < 0.05)

To further explore the relationship between ABA and anthocyanins, ABA or its biosynthetic inhibitor, fluridon (Flu), was sprayed onto the surface of *Lycium* fruits at the S1 stage, and their anthocyanin contents were measured. Compared with the control (0.49 ± 0.04 mg·g^− 1^FW), the anthocyanin concentration was increased by 27.81% in the mature fruits (S5) of YF after ABA treatment for 15 d (0.62 ± 0.04 mg·g^− 1^FW) and was decreased by 14.09% after treatment with Flu (0.42 ± 0.04 mg·g^− 1^FW) (Fig. 3a). Similarly, the application of exogenous ABA increased the anthocyanin concentration in BF from 6.14 ± 0.40 mg·g^− 1^ FW at the S1 stage to 7.77 ± 0.50 mg·g^− 1^ FW at the S5 stage (26.65% increase), whereas Flu decreased the BF anthocyanin concentration by 34.58% (4.02 ± 0.11 mg·g^− 1^ FW) compared with the control (Fig. 3b).

**Fig. 4:**
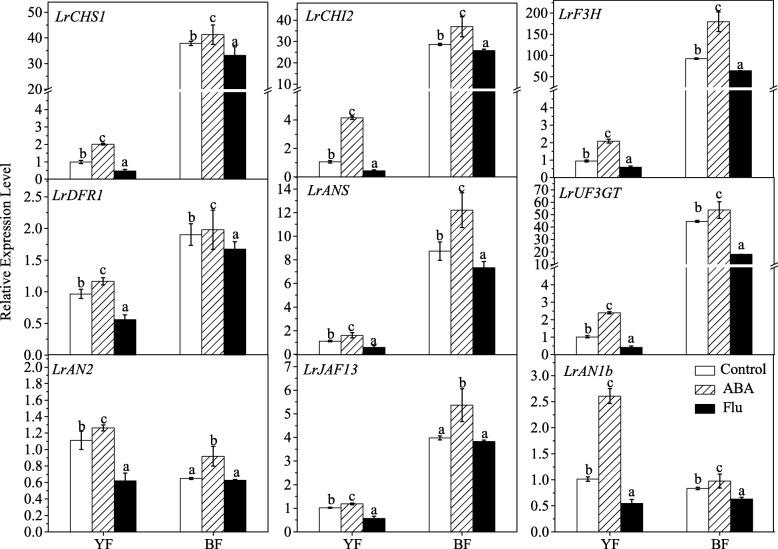
Effect of exogenous ABA or ABA biosynthesis inhibitor Flu on the transcripts of genes involved in the flavonoid biosynthetic pathway. The structural genes of *LrCHS1*, *LrCHI2, LrF3H*, *LrDFR1*, *LrANS* and *LrUF3GT*, as well as the MYB-bHLH-WD40 transcription factors, *LrAN2*, *LrJAF13* and *LrAN1b* were shown. The gene expression differences among the varieties were compared using YF (sprayed with ddH_2_O) as the control for calculating the relative expression levels of the indicated genes. The error bars represent the SDs of three independent replicates. Different letters on the bars for the same variety indicate significant differences between the treatments (P < 0.05)

ABA treatment significantly upregulated the expression of structural genes involved in the flavonoid biosynthetic pathway (Fig. 4), e.g., *LrCHS1* (chalcone synthase 1b, KC794742), *LrCHI2* (chalcone isomerase, KF031377), *LrF3H* (flavanone 3-hydroxylase, KC794744), *LrDFR1* (dihydroflavonol-4-reductase-like, KF031379)*, LrANS* (anthocyanidin synthase, KC794745), and *LrUF3GT* (UDP glucose flavonoid 3-glucosyl transferase, KF768073), which have been isolated from *Lycium ruthenicum* [27]*.* In contrast, these gene transcripts were significantly decreased after treatment with Flu. It has been reported that anthocyanin biosynthesis is regulated by the MYB-bHLH-WD40 protein complex at the transcriptional level [5]. Therefore, we further detected the transcript levels of these transcriptional factors, which have been isolated from *Lycium* plants [27]. Similar to the findings obtained for structural genes, the application of ABA also significantly upregulated the expression of regulatory genes, including *LrAN2* (anthocyanin 2, KF768075) in the R2R3 MYB family, *LrJAF13* (KF768076) and *LrAN1b* (anthocyanin 1b, KF768077) in the bHLH family [27], whereas Flu treatment significantly downregulated the expression of these genes (Fig. 4).

### Silencing of the *LbNCED1* gene suppresses *Lycium* fruit coloration

**Fig. 5:**
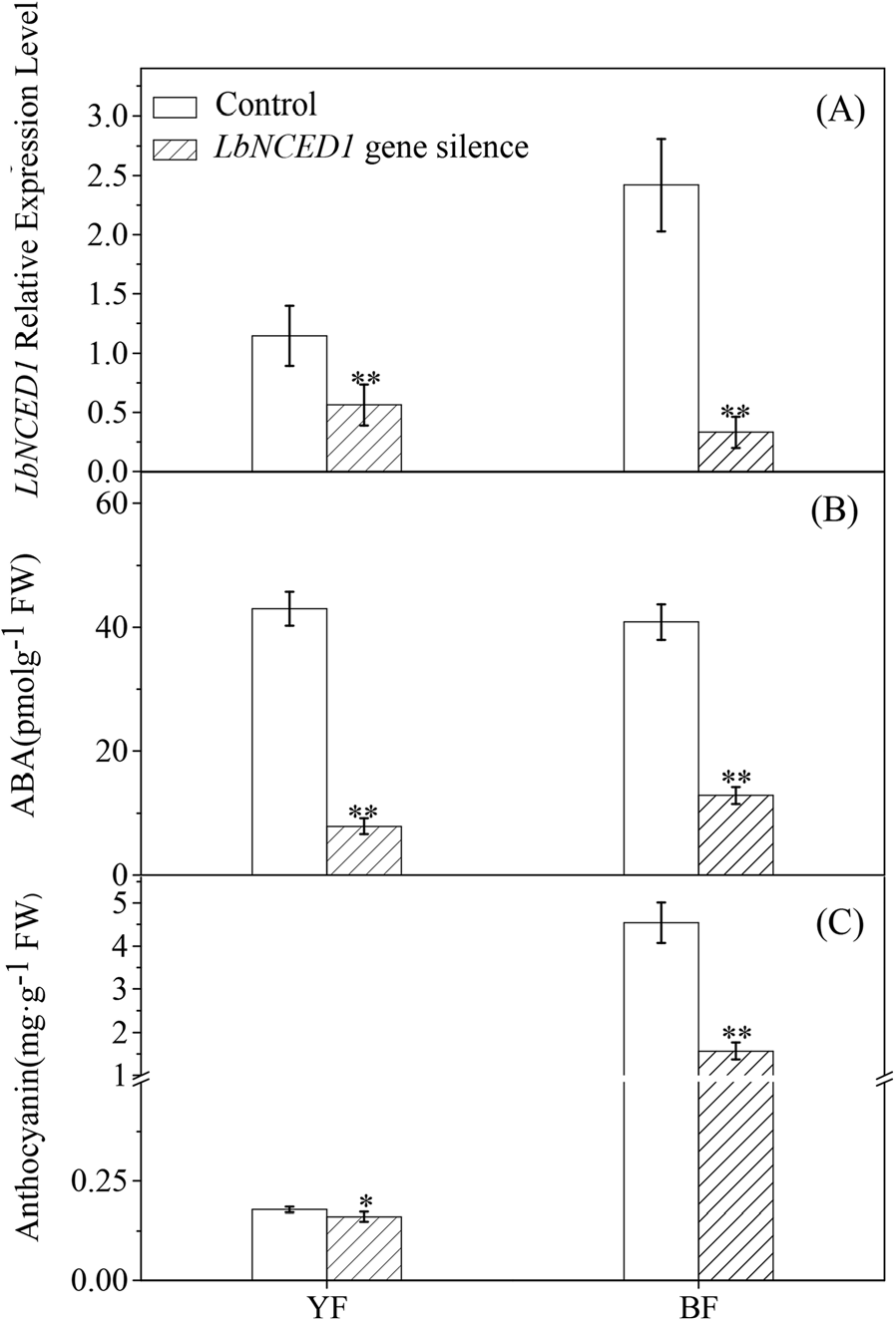
Effect of virus-induced *LbNCED1* gene silencing on ABA accumulation (**a**), anthocyanin concentration (**b**), and *LbNCED1* gene expression (**c**) in *Lycium* fruits. The data show the means ± SDs (*n* = 3). The asterisks on the bars for the same variety indicate significant differences between the treatments. “*” indicates P < 0.05, and “**” indicates P < 0.01

To further explore the effect of ABA on *Lycium* fruit ripening at the molecular genetic level, we constructed fruits with virus-induced gene silencing (VIGS) of *LbNCED1*. The expression levels of *LbNCED1* in YF and BF after VIGS were only 49.12 and 13.74% of their control values, respectively (Fig. 5a). In addition, *LbNCED1* gene silencing decreased the ABA content in the fruits of YF and BF by 81.65 and 68.43%, respectively, compared with their control values (Fig. 5b). These results indicated that our VIGS construction was credible and that the *LbNCED1* gene was successfully silenced in these two types of *Lycium* fruits. As shown in Fig. 5c, *LbNCED1* gene silencing significantly decreased the anthocyanin content by 10.48 and 65.48% in the YF and BF fruits, respectively.

**Fig. 6:**
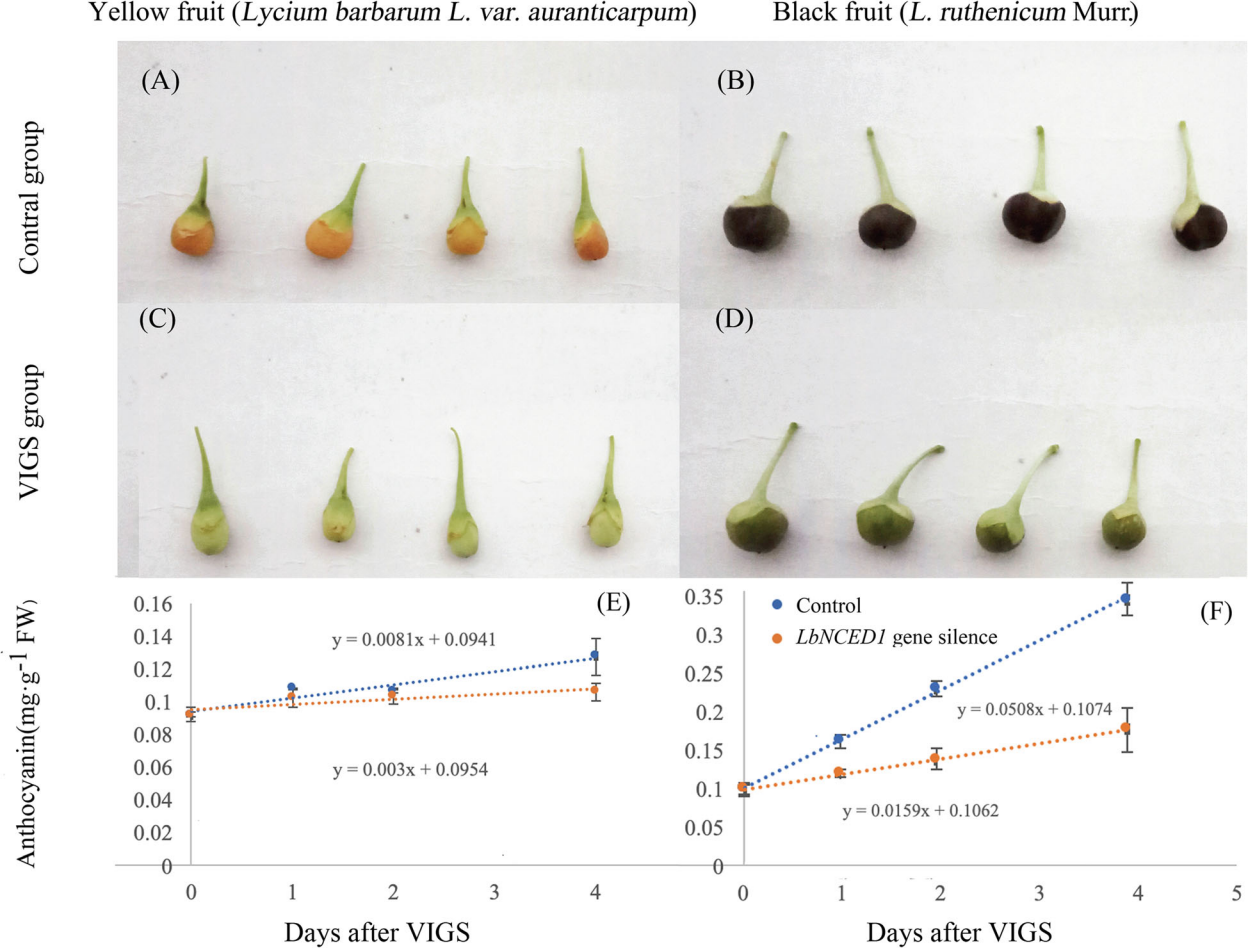
Phenotype and coloration rate of the *Lycium* fruits after virus-induced *LbNCED1* gene silencing. Representative pictures of fruits after VIGS injection for 6 d are shown in the fig. (**a** and **b**) Fruit phenotype of YF and BF after injection of the pTRV empty vector, respectively. (**c** and **d**) Fruit phenotypes of YF and BF after *LbNCED1* gene silencing, respectively. (E and F) Time-course of the anthocyanin level of YF and BF within 4 d after VIGS injection, respectively. The slope of the regression line represents the coloration rate of the fruit

The comparison of the fruit-ripening process in the control group (Fig. 6a, b) with the VIGS group (Fig. 6c, d) revealed that *LbNCED1* silencing significantly delayed fruit coloration. It is shown that whether for YF or BF, after 4 days injection, the content of anthocyanins were growing in a straight line. However, from the day 4 to day 6, anthocyanin content increase exponentially (data not shown). Therefore, the change rate of anthocyanin in the original 4 days was utilized as the coloration rate (CR, mg·g^− 1^FW·d^− 1^) of *Lycium* fruit.We found that *LbNCED1* gene silencing significantly reduces CR for both of the fruits, in which the CR of YF fell from 0.008 mg·g^− 1^FW·d^− 1^ to 0.003 mg·g^− 1^FW·d^− 1^ (Fig. 6e), while the BF fell from 0.051 mg·g^− 1^FW·d^− 1^ to 0.015 mg·g^−1^FW·d^−1^ ((Fig. 6f).

### Silencing of the *LbNCED1* gene alters the expression of genes involved in anthocyanin biosynthesis

**Fig. 7:**
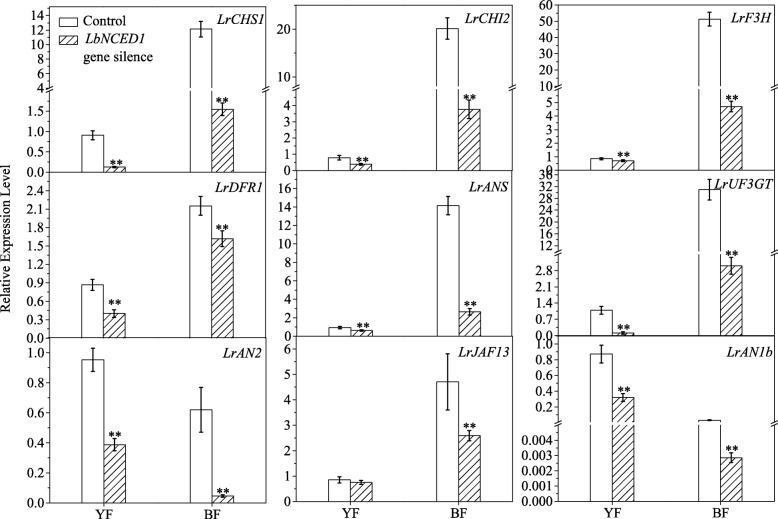
Effect of *LbNCED1* gene silencing on the transcripts of genes involved in the flavonoid biosynthetic pathway. The structural genes of *LrCHS1*, *LrCHI2, LrF3H*, *LrDFR1*, *LrANS* and *LrUF3GT*, as well as the MYB-bHLH-WD40 transcription factors, *LrAN2*, *LrJAF13* and *LrAN1b* were shown. The gene expression differences among the varieties were compared using YF (injected with empty vector) as the control for calculating the relative expression levels of the indicated genes. The data show the means ± SDs (n = 3). The asterisks on the bars for the same variety indicate significant differences between the treatments. “*” indicates P < 0.05, and “**” indicates P < 0.01

To explore the mechanism underlying the effect of LbNCED-derived ABA on anthocyanin biosynthesis during *Lycium* fruit ripening, the transcript amounts of flavonoid biosynthetic pathway-related genes in the VIGS-modified fruits was determined by qRT-PCR technology. *LbNCED1* silencing significantly downregulated the expression of structural genes involved in this pathway, e.g., *LrCHS1*, *LrCHI2, LrF3H*, *LrDFR1, LrANS,* and *LrUF3GT* (Fig. 7)*.* In YF and BF, the extent of the decreases in the gene transcript levels ranged from 17.57 to 84.96% and 24.86 to 90.83%, respectively. Thus, BF showed a more substantial decrease compared with YF. In addition to this genetic variation, a gene-specific sensitivity to endogenous ABA was also observed among these structural genes involved in the flavonoid biosynthesis pathway in *Lycium* fruits.

Similar to the structural genes, *LbNCED1* silencing also significantly downregulated the expression of regulatory genes (Fig. 7), including *LrAN2*, *LrJAF13* and *LrAN1b*. In YF and BF, the levels of these genes decreased in the ranges of 11.47 to 63.36% and 69.60 to 92.45%, respectively. In addition, the transcript amounts of the transcriptional factors related to *LrAN2, LrJAF13* and *LrAN1b* decreased in the ranges of 59.31 to 92.45%, 11.47 to 69.60%, and 21.07 to 90.99%, respectively*.* These results indicated that the changing trends of these regulator genes were similar to those of the structural genes in *Lycium* varieties under *LbNCED1* silencing and corresponded to their distinct anthocyanin levels. Moreover, the transcriptional response of these transcriptional factors to endogenous ABA showed some degree of diversity.

## Discussion

Because fleshy fruits become attractive and nutritious to seed-dispersing animals, the transition from unripe to ripe fruit represents a dramatic shift in the survival strategy of plants [1]. During the process of fruit ripening, secondary metabolites are accumulated, and anthocyanins, which are one of the pigments, increase, resulting in fruit coloration [6]. Similarly, the ABA level also increases during fruit growth and maturation [8]. In the current study, we used *Lycium* fruit as a model to shown a strong positive correlation between the ABA levels and the anthocyanin content. These results imply that the relationship between ABA and anthocyanins is well conserved in both fruit trees, including those with climacteric and nonclimacteric fruits, and vegetables during fruit ripening. Because the baseline value of the anthocyanin content in RF (as the stable cultivar) is very low, the effect of ABA treatment is not obvious (data not shown). Given that the dominant pigment in RF is carotenoid [27], the mechanism through which ABA mediates carotenoid biosynthesis in RF should be urgently addressed in the future.

Our results indicated that anthocyanin levels in *Lycium* fruits were affected both by developmental stage and genotypes. Whereas the variety-specific difference may primarily be determined by geographic variables (latitude, elevation) of the source area, the development-dependent variation may be affected by endogenous phytohomones [3, 5]. In China, the wild BF is mainly located in Qaidam Basin (in Qinghai province, mean altitude: 2700 m), while the wild YF is mainly found in Yinchuan suburb (in the Ningxia Hui Autonomous Region, mean altitude: 1100 m) [28]. Relative to the low altitude areas, there is a high light intensity, long sunshine time, strong UV-B and low temperature in the high altitude areas. These environmental factors have been shown to be beneficial to anthocyanin biosynthesis [5]. Plant hormones have a crucial role in the regulation of fruit development and ripening, and thus have a potential effect in anthocyanin biosynthesis [3, 5]. The present work demonstrated that the ABA level of *Lycium* fruit kept on rising during ripening, however, other reports have exhibited lack of an Eth burst during this process [30], indicating the *Lycium* fruit belongs to non-climacteric fruit. The crosstalk of ABA with other phytohormones, e.g., JA, auxin, GA, and cytokinin is known to promote or inhibit the biosynthesis of anthocyanin in other fruits [5], which should be addressed in the future in *Lycium* fruits. Moreover, because fruit ripening is generally accompanied with the senescence, *reactive oxygen species* are also involved in fruit maturation, irrespective of climacteric or non-climacteric fruits [32]. How these small molecules coordinate and interact with ABA to control the ripening process of *Lycium* fruits also needs to be highlighted.

NCED is generally encoded by a small gene family [14]. Among the three NCED genes in tomato, *SlNCED1* might play a major role in regulating ABA biosynthesis in response to ABA application and dehydration during fruit ripening [15]. The silencing of *VmNCED1*, the key gene in ABA biosynthesis in ripening bilberry, is accompanied by downregulation of the expression of key anthocyanin biosynthesis-related genes [24]. In this study, we used VIGS technology to silence the ABA biosynthesis-related gene *LbNCED1* in *Lycium* fruit to further verify the relationship between ABA function and fruit coloration during fruit ripening. The *LbNCED1*-RNAi-treated fruit showed similarity in the results reported by Ji et al. [15], in which the ripening of fruit was inhibited and also complete coloration and softening did not occur. Based on this finding, it can be concluded that unlike the control fruit, the VIGS-modified fruit did not undergo the normal maturation process. All the above-described data indicate that the *LbNCED1* gene plays a positive regulatory role in *Lycium* fruit ripening by mediating ABA biosynthesis and anthocyanin accumulation. ABA is also closely related to fruit color regulation and pigment synthesis at the molecular genetic level.

Fruits have become a good system for studying anthocyanin biosynthesis regulation by the MYB-bHLH-WD40 transcription factor complex, as has been shown in tomato, apple, sweet cherry, muscadine berry, strawberry and pear plants [33–38]. In the present work, the transcripts of this type of transcription factor showed a similar changing trend as the structural genes and anthocyanin content following *LbNCED1* gene silencing in *Lycium* fruits. It can be concluded that NCED-derived ABA transcriptionally regulates the transcription factors *LbAN2*, *LbJAF13* and *LbAN1b* in *Lycium* fruits, and these transcription factors activate anthocyanin biosynthesis-related gene expression and enhance anthocyanin generation. These ABA-mediated anthocyanin biosynthesis pathways have been recently validated in sweet cherry [34] and apple plants [21]. Future studies should precisely explore which of the pathways are responsible for regulating the early biosynthesis-related genes that are common to the different flavonoid subpathways or the late genes that are specific for the anthocyanins or proanthocyanin subpathways, as has been shown in *Arabidopsis* [37].

As flavonoid compounds, anthocyanins and proanthocyanins have a strong antioxidant capacity [5]. Therefore, anthocyanins are not only a determinant of fruit color but also a sign of fruit quality, particularly for fruits that are utilized for both medicine and food. In this case, the regulation of fruit color also corresponds to the regulation of fruit quality. It is well known that ABA is a stress phytohormone, and its level can be finely tuned by environmental factors, particularly a water deficit [14]. Therefore, controlling the water deficit during fruit ripening can affect both anthocyanin synthesis and fruit quality by regulating endogenous ABA homeostasis, as has been shown in the Cabernet Sauvignon grape [39]. In addition, the implication of artificial synthetic ABA or ABA analogs on fruit ripening and quality modifications is another promising direction in practice [17, 18], as has been observed in the response of crop plants to drought stress [40]. The molecular mechanisms underlying *Lycium* fruit coloration and ripening regulation by environmental factors should be addressed in the future. Interestingly, *Lycium* fruits might be a potential model system for studying the geoherbalism of traditional Chinese medicines due to their color diversity and clear phenotype changes.

## Conclusions

**Fig. 8:**
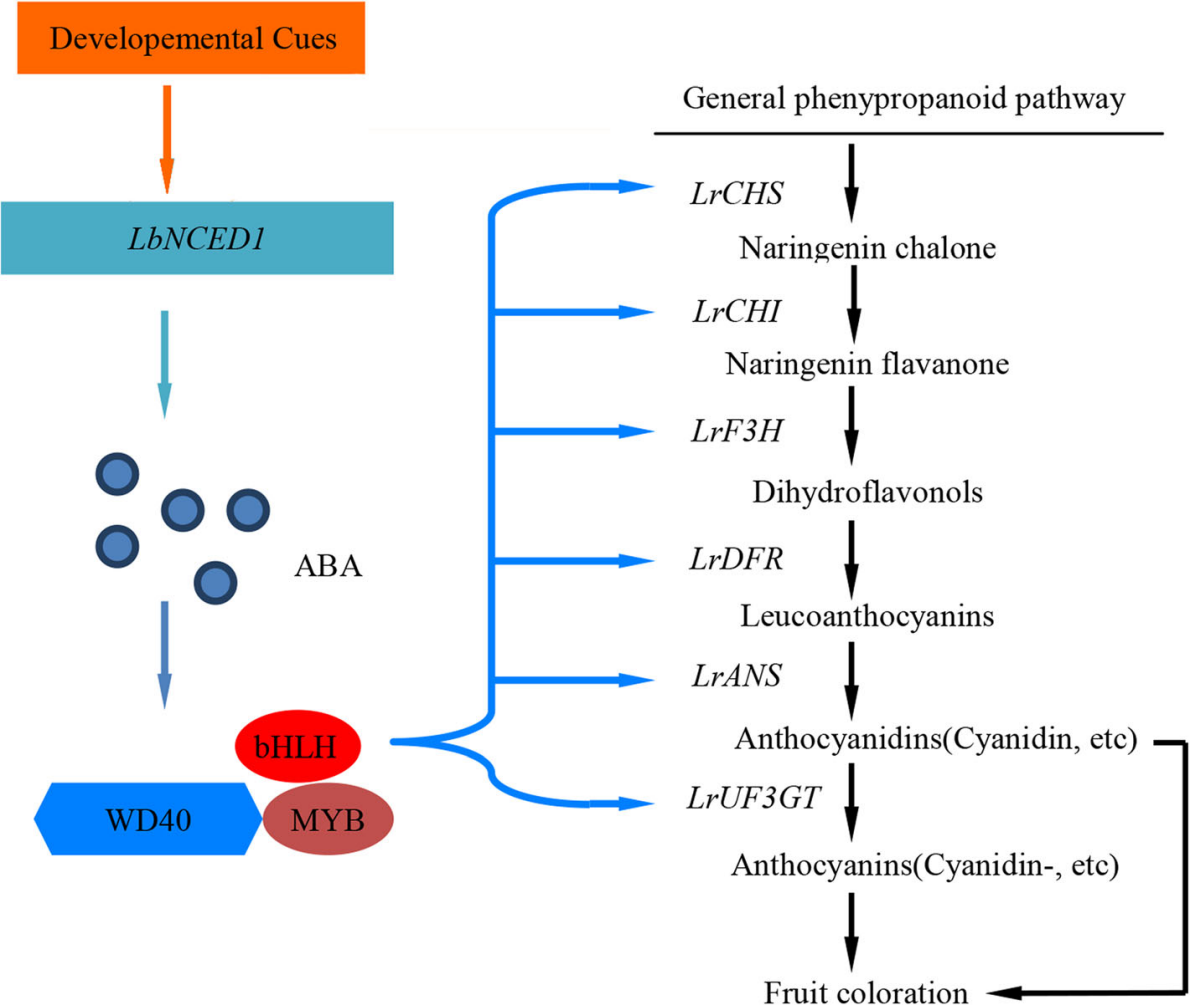
Model of ABA-regulated anthocyanin biosynthesis during *Lycium* fruit ripening. The developmental cues transcriptionally activates *LbNCED1* and thus enhances endogenous ABA accumulation. This accumulation is followed by the transcriptional stimulation of the MYB-bHLH-WD40 transcription factor complex, upregulation of the expression of structural genes involved in the flavonoid biosynthetic pathway, and finally promotion of anthocyanin production and fruit coloration

In this study, a model was constructed for ABA-mediated development-dependent anthocyanin biosynthesis and fruit coloration during *Lycium* fruit maturation. In this model, the developmental cues transcriptionally activated *LbNCED1* and thus enhanced the accumulation of the phytohormone ABA. This step was followed by the transcriptional stimulation of the MYB-bHLH-WD40 transcription factor complex, upregulation of the expression of structural genes involved in the flavonoid biosynthetic pathway, and finally promotion of anthocyanin production and fruit coloration (Fig. 8). Our results might have filled the gap between developmental cues and transcription factors in the regulation of anthocyanin biosynthesis and provide valuable information for improving both the nutritional quality and the pharmaceutical value of *Lycium* fruits through ABA signaling engineering.

## Methods

### Plant material

*Lycium* fruits of two different colors, namely, black fruit (Heiguo, *Lycium ruthenicum* Murr. First identified by Wang Y. Z., and the voucher sample is now deposited in the Herbarium of the Institute of Botany, Chinese Academy of Sciences. Deposition number: PE 00672229), and yellow fruit (Huangguo, *Lycium barbarum L. var. auranticarpum.* First identified by Ching K.F., and the voucher sample is now deposited in the Herbarium of the Institute of Botany, Chinese Academy of Sciences. Deposition number: PE 00031412), were collected from two types of 5-year-old trees at the Wolfberry (*Lycium*) Germplasm Repository of Ningxia, Academy of Agriculture and Forestry Sciences, Ningxia Hui Autonomous Region, China (38°380 N, 106°090 E and altitude 1100 m). Our field studies were conducted in accordance with local legislation and appropriate permissions.

For the assaying of fruit ripening dynamics, fruits were sampled at five ripening stages (S1-S5) in their natural state, as described by Zeng et al. [27], with some minor modifications. The ripening process was divided in detail into the immature green fruit stage of both varieties (S1, 9 d after anthesis (DAA)); the yellowish fruit stage of YF and the pale pink fruit stage of BF (S2, 14 DAA); the successive ripening fruit stage of both varieties (S3, 20 DAA); the stage associated with the deepening of fruit pigmentation in both varieties (S4, 30 DAA); and the stage of full fruit ripening with full expansion, which was characterized by a mature black fruit in BF and a mature yellow fruit in YF (S5, 34 DAA). All fruit samples were frozen in liquid nitrogen immediately after harvesting and stored at − 80 °C until further analysis.

### Exogenous hormone and hormone inhibitor treatment

To avoid strong transpiration, the treatment was performed before evening. The surfaces of immature healthy fruits (at the S1 stage) were sprayed with ABA (50 mg·L^− 1^) or the ABA biosynthesis inhibitor fluoridone (Flu, 50 μM), and the control was sprayed with ddH_2_O (including 0.05% Tween 20). The reagent was diluted with ddH_2_O containing 0.05% Tween 20 to ensure increased adhesion on the blade surface. Our experimental design was based on a randomized complete block design (RCBD). Each treatment ensured three trees with a similar growth conditions, and a total of nine trees were needed per variety. The whole tree or some branches within one tree were sprayed according to their amount of fruits. For each *Lycium* variety, the reagent was sprayed on at least 200 healthy fruits per tree to ensure that the fruits were 100% soaked and to ensure that the fruits were free from disease, insect and mechanical damage. At least 600 healthy fruits in total were included in each treatment per variety. The fruits were sampled 15 d after spraying, frozen immediately in liquid nitrogen and stored at − 80 °C until further analysis.

### Virus-induced gene silencing (VIGS) vector construction

The *LbNCED1* (KF957694.1) gene was screened from the NCBI database. The CDS sequence of the *LbNCED* gene was amplified with the following primers: 5′-CGACGACAAGACCCT-ATGGCCACTTCTTCTCCTGCTAC-3′ and 5′-GAGGAGAAGAGCCCT-TTAGGCCTGATTTGCCAAGTCTT-3′. The PCR products were purified with a TIANquick Midi Purification Kit (DP204, TianGen, Beijing, China). A total of 50 ng of purified PCR product was treated with T4 DNA polymerase in a 10× T4 DNA polymerase buffer containing 5 mM dATP and 0.1 M dithiothreitol at 22 °C for 30 min, and T4 DNA polymerase was then inactivated for 20 min at 75 °C. The TRV2-LIC vector was then digested with PstI and similarly treated with T4 DNA polymerase, with the exception that dTTP was used instead of dATP. The treated PCR product was mixed with the vehicle in an equal volume and incubated at 22 °C for 10 min for ligation.Then, 10 μL of the mixture was transformed into *E. coli* DH5α competent cells. Transformants were tested by PCR amplification using the primers 5′-ATGGCCACTTCTTCTCCTGCTAC-3′ and 5′-TTAGGCCTGATTTGCCAAGTCTT-3′. The plasmids from the positive clones were purified and sequenced.

### VIGS silencing vector expression

For VIGS TRV1 [41], TRV2, TRV2-LIC or its derivatives was introduced into *Agrobacterium tumefaciens* strain GV3101 by heat shock. For screening the TRV2-LIC collection, 25-mL overnight cultures were grown at 28 °C in the presence of the appropriate antibiotics (50 mg·L^− 1^ rifampicin and 50 mg·L^− 1^ kanamycin). The next day, the cultures were centrifuged, and the cells were resuspended in the infiltration medium (10 mM MES, 10 mM MgCl_2_, and 200 μM acetosyringone). The OD_600_ was adjusted to 1.0, and the cells were incubated at room temperature for 3~4 h. Subsequently, the pTRV1 and pTRV2 (or pTRV2-LIC) solutions were mixed at a ratio of 1:1 for injection.

The injection was performed as described by Dong et al. [42], with some minor modifications. Briefly, immature fruits (at the S1 stage) were injected at the fruit growth point (located at the fruit tail) using a 1-mL syringe. The injected amount depended on the degree of diffusion of the agrobacterium solution in the fruits, and the fruits that were successfully injected were bright and easily distinguished. The empty carrier was injected as the control. Each vector was injected into fruits from at least three trees of each variety and at least 200 healthy fruits per tree. The proportion of the fruit color change was determined within 6 d after the injection, and the samples were collected after 6 d. All fruit samples were frozen in liquid nitrogen immediately after harvesting and stored at − 80 °C until further analysis.

### ABA extraction and assay

Endogenous ABA was extracted from the fruits according to the method described by Fan et al. [43], with some minor modifications. Briefly, 0.2 g of fruit sample was fully ground with 1 mL of precooled 80% methanol extract solution (containing 200 mg·L^− 1^ 2,6-di-tert-butyl-p-cresol and 500 mg·L^− 1^ citric acid monohydrate). After overnight leaching at 4 °C, the mixture was centrifuged at 10,000 rpm and 4 °C for 15 min, and the above-described procedure was repeated with the supernatant. The twice-treated supernatant was then combined, and the ground fruit was concentrated and dried with a Visible Nitrogen Blower (KD200, ALLSHENG, China). Finally, 0.8 mL of precooled 80% methanol was added to the dry powder, and the mixture was mixed with a vortex shaker to form the crude extract of ABA.

ABA was assayed using a Phytodetek Immunoassay Kit (PDK 09347/0096, Agdia, USA) according to the manufacturer’s instructions. First, the crude ABA extract was diluted 11 times with TBS buffer (1:10 volume ratio of ABA crude extract to TBS buffer). The plate was prepositioned for 15 min at room temperature, and 100 μL of the solution was added to the antibody-labeled plate. An equal volume of diluted tracer was rapidly added, and the plate was then mixed well and incubated at 4 °C for 3 h. After the incubation, the reaction solution in the microplate was quickly poured off. After blotting with filter paper (and ensuring that no liquid remained), the micropores were subjected to two 30-s rinses with 1× PBST, and 200 μL of the substrate reaction solution (freshly prepared and thoroughly mixed before use) was added to the micropores and bathed at 37 °C for 1 h. Note that this procedure should be performed in the dark. The absorbance at 405 nm was measured, and the ABA concentration was calculated from the standard curve.

### Anthocyanin determination

The fruit powder was quickly weighed (0.2 g) and placed in a 1.5-mL centrifuge tube. After the addition of 1 mL of anthocyanin extract solution (1% hydrochloric acid in methanol), the tube was shaken and placed in a refrigerator at 4 °C for overnight extraction (more than 16 h) [44]. Following centrifugation for 10 min at 2500×g, 400 μL of supernatant was transferred to another 1.5-mL centrifuge tube, and 400 μL of chloroform and dH_2_O were added. After centrifugation at 2500×g for 10 min, 600 μL of the supernatant was pipetted to another 1.5-mL centrifuge tube, and the absorbance value at 530 nm was measured using a Microplate Reader (Spectra Max M2, Molecular Devices, USA). The anthocyanin concentration was calculated according to the standard curve, which was obtained using cyanidin-3-glucoside (626B021, Solarbio, China) as the standard. Three biological replicates of each variety and each treatment were tested.

### Determination of the fruit coloration rate

The changes of fruit anthocyanin level were determined at 0, 1, 2, 4 and 6 day after injection. The fruit coloration rate was reported as milligram anthocyanin (mg) per gram fresh weight fruit (g^− 1^FW) per day (d^− 1^).

### Quantitative reverse transcription-PCR (qRT-PCR)

The total RNA from the samples was extracted using an RNA prep Pure Plant Kit (DP432, TianGen, Beijing, China), and the cDNA was synthesized through reverse transcription using a Fasting cDNA First-Strand Synthesis Kit (TianGen, Beijing, China) according to the manufacturer’s instructions. For the relative gene expression assay, the housekeeping gene β-actin [45] was employed as an internal control because it is assumed that this gene exhibits uniform expression in *Lycium* [27]. The qRT-PCR procedure was performed using a BioRad CFX96 Real-Time PCR Detection System (USA) with a qRT-PCR kit (TianGen, Beijing, China) according to the manufacturer’s recommendations. Each 20-μL reaction contained 10 μL of 2x SuperReal Color PreMix (SYBR Green), 0.8 μL of forward primer (10 μM), 0.8 μL of reverse primer (10 μM), 1.5 μL of cDNA template (20 ng) and 6.9 μL of H_2_O. The following three-step assay conditions for qRT-PCR were designed and tested: one cycle of 95 °C/15 min followed by 40 cycles of 95 °C/10 s, 55 °C/30 s, and 72 °C/32 s. The data were analyzed using the 2^–ΔΔCt^ method [46]. The relative expression levels are expressed as the means ± SDs of three replicates.

The primers used for qRT-PCR were the following: *Actin1*-F, GGAAACATAGTGCTCAGTGGTG; *Actin1-*R, GCTGAGGGAAGCCAAGATAG; *LbNCED1-*F, GCAGCAGCAATGGCTTTAGA, *LbNCED1-*R, GGTGACCGGAAGTGATTGAGT; *LrANS-*F, GATCCACCTCGATTCCCACC; and *LrANS-*R, TGTTCATCCTTTTTGGCGGC. The primers for the structural genes *LrCHS1*, *LrCHI2*, *LrF3H*, *LrUF3GT* and *LrDFR1* and for the transcription factors *LrAN2*, *LrJAF13*, and *LrAN1b* were designed as described by Zeng et al. [27].

### Statistical analysis

A statistical analysis using Duncan’s multiple range test (IBM SPSS Statistics 19.0) was used to assess the significance of the differences between two samples. Differences between the means were considered significant if the *P*-value obtained from the ANOVA was less than 0.05. The significance of the differences between two samples was analyzed by a t-test (“*” indicates P-value < 0.05; “**” indicates P-value < 0.01). The Pearson correlation coefficient was used to evaluate the relationship among the ABA level, anthocyanin level and *LbNCED1* transcript abundance during fruit ripening. The bar and line charts were plotted using Origin 9.0.

## Data Availability

The data sets supporting the results of this article are included within the article. Ethics approval and consent to participate. The fruit samples were collected with the consent of the Ningxia Academy of Agriculture and Forestry Sciences, China. No other permissions were necessary to collect these samples.

## Abbreviations

ABA: Abscisic acid
AN: Anthocyanin
ANS: Anthocyanidin synthase
CHI: Chalcone isomerase
CHS: Chalcone synthase
DFR: Dihydroflavonol-4-reductase-like
F3H: Flavanone 3-hydroxylase
NCED: 9-cis-epoxycarotenoid dioxygenase
qRT-PCR: Quantitative reverse transcription-PCR
UF3GT: UDP-glucose flavonoid 3-glucosyl transferase
VIGS: Virus-induced gene silencing

## References

[1] Giovannoni J, Nguyen C, Ampofo B, Zhong S, Fei Z (2017). The epigenome and transcriptional dynamics of fruit ripening. Ann Rev Plant Biol.

[2] Jia H, Jiu S, Zhang C, Wang C, Tariq P, Liu Z (2016). Abscisic acid and sucrose regulate tomato and strawberry fruit ripening through the abscisic acid-stress-ripening transcription factor. Plant Biotechnol J.

[3] Petroni K, Tonelli C (2011). Recent advances on the regulation of anthocyanin synthesis in reproductive organs. Plant Sci.

[4] Khoo HE, Azlan A, Tang ST, Lim SM (2017). Anthocyanidins and anthocyanins: colored pigments as food, pharmaceutical ingredients, and the potential health benefits. Food Nutr Res.

[5] Jaakola L (2013). New insights into the regulation of anthocyanin biosynthesis in fruits. Trends Plant Sci.

[6] Jia HF, Chai YM, Li CL, Lu D, Luo JJ, Qin L (2011). Abscisic acid plays an important role in the regulation of strawberry fruit ripening. Plant Physiol.

[7] Rafique MZ, Carvalho E, Stracke R, Palmieri L, Herrera L, Feller A (2016). Nonsense mutation inside anthocyanidin synthase gene controls pigmentation in yellow raspberry (*Rubus idaeus* L.). Front Plant Sci.

[8] Teribia N, Tijero V, Munne-Bosch S (2016). Linking hormonal profiles with variations in sugar and anthocyanin content during the natural development and ripening of sweet cherries. New Biotech.

[9] Oglesby L, Ananga A, Obuya J, Ochieng J, Cebert E, Tsolova V (2016). Anthocyanin accumulation in muscadine berry skins is influenced by the expression of the MYB transcription factors, MybA1, and MYBCS1. Antioxidants (Basel).

[10] Zhai R, Wang Z, Zhang S, Meng G, Song L, Wang Z (2016). Two MYB transcription factors regulate flavonoid biosynthesis in pear fruit (Pyrus bretschneideri Rehd.). J Exp Bot.

[11] Lu Y, Bu Y, Hao S, Wang Y, Zhang J, Tian J (2017). MYBs affect the variation in the ratio of anthocyanin and flavanol in fruit peel and flesh in response to shade. J Photochem Photobiol B.

[12] Murcia G, Fontana A, Pontin M, Baraldi R, Bertazza G, Piccoli PN (2016). ABA and GA3 regulate the synthesis of primary and secondary metabolites related to alleviation from biotic and abiotic stresses in grapevine. Phytochem..

[13] Olivares D, Contreras C, Munoz V, Rivera S, Gonzalez-Aguero M, Retamales J (2017). Relationship among color development, anthocyanin and pigment-related gene expression in ‘Crimson Seedless’ grapes treated with abscisic acid and sucrose. Plant Physiol Biochem.

[14] Finkelstein R (2013). Abscisic acid synthesis and response. The Arabidopsis Book.

[15] Ji K, Kai W, Zhao B, Sun Y, Yuan B, Dai S (2014). SINCED1 and SICYP07A2: key genes involved in ABA metabolism during tomato fruit ripening. J Exp Bot.

[16] Liao X, Li M, Liu B, Yan M, Yu X, Zi H, Liu R, Yamamuro C (2018). Interlinked regulatory loops of ABA catabolism and biosynthesis coordinate fruit growth and ripening in woodland strawberry. Proc Natl Acad Sci U S A.

[17] Tijero V, Teribia N, Munoz P, Munne-Bosch S (2016). Implication of abscisic acid on ripening and quality in sweet cherries: differential effects during pre- and post-harvest. Front Plant Sci.

[18] Miret JA, Munne-Bosch S (2016). Abscisic acid and pyrabactin improve vitamin c contents in raspberries. Food Chem.

[19] Symons GM, Chua YJ, Ross JJ, Quittenden LJ, Davies NW, Reid JB (2012). Hormonal changes during non-climacteric ripening in strawberry. J Exp Bot.

[20] Villalobos-Gonzalez L, Pena-Neira A, Ibanez F, Pastenes C (2016). Long-term effects of abscisic acid (ABA) on the grape berry phenylpropanoid pathway: gene expression and metabolite content. Plant Physiol Biochem.

[21] An JP, Yao JF, Xu RR, You CX, Wang XF, Hao YJ (2018). Apple bZIP transcription factor MdbZIP44 regulates abscisic acid-promoted anthocyanin accumulation. Plant Cell Environ.

[22] Oh HD, Yu DJ, Chung SW, Chea S, Lee HJ (2018). Abscisic acid stimulates anthocyanin accumulation in ‘jersey’ highbush blueberry fruits during ripening. Food Chem.

[23] Zhang Y, Li Q, Jiang L, Kai W, Liang B, Wang J (2018). Suppressing type 2C protein phosphatases alters fruit ripening and the stress response in tomato. Plant Cell Physiol.

[24] Karppinen K, Tegelberg P, Haggman H, Jaakola L (2018). Abscisic acid regulates anthocyanin biosynthesis and gene expression associated with cell wall modification in ripening bilberry (*Vaccinium myrtillus* L.) fruits. Front Plant Sci.

[25] Zhao J, Li H, Xi W, An W, Niu L, Cao Y, Wang H, Wang Y, Yin Y (2015). Changes in sugars and organic acids in wolfberry (*Lycium barbarum* L.) fruit during development and maturation. Food Chem.

[26] Potterat O (2010). Goji (*Lycium barbarum* and *L. chinense*): phytochemistry, pharmacology and safety in the perspective of traditional uses and recent popularity. Planta Med.

[27] Zeng S, Wu M, Zou C, Liu X, Shen X, Hayward A (2014). Comparative analysis of anthocyanin biosynthesis during fruit development in two lycium species. Physiol Plant.

[28] Yao R, Heinrich M, Weckerle CS (2018). The genus *Lycium* as food and medicine: a botanical, ethnobotanical and historical review. J Ethnopharmacol.

[29] Liu Y, Zeng S, Sun W, Wu M, Hu W, Shen X, et al. Comparative analysis of carotenoid accumulation in two goji (*Lycium barbarum* L. and *L. ruthenicum* Murr.) fruits. BMC Plant Biol. 2014;14: 269. doi: 10.1186. 10.1186/s12870-014-0269-4 PMC4276078 25511605

[30] Mi J, Lu L, Dai G, He X, Li X, Yan Y (2018). Correlations between skin color and carotenoid contents in wolfberry. Food Sci.

[31] Tian X, Ji J, Wang G, Jin C, Guan C, Wu D, Li Z (2015). Cloning and expression analysis of 9-cis-epoxycarotenoid dioxygenase gene 1 involved in fruit maturation and abiotic stress response in Lycium chinense. J Plant Growth Regul.

[32] Gapper NE, McQuinn RP, Giovannoni JJ (2013). Molecular and genetic regulation of fruit ripening. Plant Molecul Biol.

[33] Schaart JG, Dubos C, Romero De La Fuente I, van Houwelingen AM, de Vos RC, Jonker HH (2012). Identification and characterization of MYB-bHLH-WD40 regulatory complexes controlling proanthocyanidin biosynthesis in strawberry (Fragaria× ananassa) fruits. New Phytol.

[34] Xie X, Li S, Zhang R, Zhao J, Chen Y, Zhao Q (2012). The bHLH transcription factor MdbHLH3 promotes anthocyanin accumulation and fruit colouration in response to low temperature in apples. Plant Cell Environ.

[35] Chagne D, Lin-Wang K, Espley RV, Volz RK, How NM, Rouse S (2013). An ancient duplication of apple MYB transcription factors is responsible for novel red fruit-flesh phenotypes. Plant Physiol.

[36] Shen X, Zhao K, Liu L, Zhang K, Yuan H, Liao X (2014). A role for PacMYBA in ABA-regulated anthocyanin biosynthesis in red-colored sweet cherry cv. Hong Deng (*Prunus avium* L.). Plant Cell Physiol..

[37] Chezem WR, Clay NK (2016). Regulation of plant secondary metabolism and associated specialized cell development by MYBs and bHLHs. Phytochemistry..

[38] Gao Y, Liu J, Chen Y, Tang H, Wang Y, He Y (2018). Tomato SlAN11 regulates flavonoid biosynthesis and seed dormancy by interaction with bHLH proteins but not with MYB proteins. Hortic Res.

[39] Caceres-Mella A, Talaverano MI, Villalobos-Gonzalez L, Ribalta-Pizarro C, Pastenes C (2017). Controlled water deficit during ripening affects proanthocyanidin synthesis, concentration and composition in cabernet sauvignon grape skins. Plant Physiol Biochem.

[40] Cao MJ, Zhang YL, Liu X, Huang H, Zhou XE, Wang WL (2017). Combining chemical and genetic approaches to increase drought resistance in plants. Nat Commun.

[41] Liu Y, Schiff M, Marathe R, Dinesh-Kumar SP (2002). Tobacco Rar1, EDS1 and NPR1/NIM1 like genes are required for N-mediated resistance to tobacco mosaic virus. Plant J.

[42] Dong Y, Burchsmith TM, Liu Y, Mamillapalli P, Dineshkumar SP (2007). A ligation-independent cloning tobacco rattle virus vector for high-throughput virus-induced gene silencing identifies roles for NbMADS4-1 and −2 in floral development. Plant Physiol.

[43] Fan W, Zhao M, Li S, Xue B, Jia L, Meng H (2016). Contrasting transcriptional response of PYR1/PYL ABA receptors to ABA or dehydration stress between maize seedlings leaves and roots. BMC Plant Biol.

[44] Pang Y, Peel GJ, Sharma SB, Tang Y, Dixon RA (2008). A transcript profiling approach reveals an epicatechin-specific glucosyltransferase expressed in the seed coat of medicago truncatula. Proc. Natl Acad Sci U S A.

[45] Wu D, Ji J, Wang G, Guan C, Jin C (2014). LchERF, a novel ethylene-responsive transcription factor from lycium chinense, confers salt tolerance in transgenic tobacco. Plant Cell Rep.

[46] Livak KJ, Schmittgen TD (2001). Analysis of relative gene expression data using real-time quantitative PCR and the 2(−Delta Delta C(T)) method. Methods.

